# Limited yield of SARS-CoV-2 screening in asymptomatic hematopoietic cell transplant and chimeric antigen receptor T-cell therapy patients

**DOI:** 10.1017/ash.2025.10112

**Published:** 2025-08-29

**Authors:** Marie Hale Wilson, Elizabeth M. Krantz, Steven A. Pergam, Marco Mielcarek, Suni Elgar, Emily Rosen, Michelle Swetky, Catherine Liu, Joshua A. Hill, Seth Cohen, Denise J. McCulloch

**Affiliations:** 1 Quality Department, Fred Hutchinson Cancer Center, 1100 Fairview Ave N., Seattle, WA, 98109, USA; 2 Vaccine and Infectious Disease Division, Fred Hutchinson Cancer Center, Seattle, WA, USA; 3 Adult Blood and Marrow Transplant Program, Fred Hutchinson Cancer Center, Seattle, WA, USA; 4 Clinical Research Division, Fred Hutchinson Cancer Center, Seattle, WA, USA; 5 Division of Hematology and Oncology, University of Washington, Seattle, WA, USA; 6 Clinical Administration, Fred Hutchinson Cancer Center, Seattle, WA, USA; 7 Division of Allergy and Infectious Diseases, University of Washington, Seattle, WA, USA

## Abstract

Early in the COVID-19 pandemic, screening was initiated in several settings to mitigate asymptomatic transmission of SARS-CoV-2. However, this practice was later discouraged by the Society for Healthcare Epidemiology of America. This single-center retrospective study demonstrates limited utility of SARS-CoV-2 screening tests in asymptomatic HCT and CAR T-cell patients.

## Introduction

Testing of asymptomatic persons (screening) for severe acute respiratory syndrome coronavirus 2 (SARS-CoV-2) was one intervention widely recommended by public health authorities and employed by health care institutions to mitigate the spread of coronavirus disease-2019 (COVID-19) during the pandemic, especially among those at high risk of morbidity and mortality from COVID-19, such as patients undergoing hematopoietic stem cell transplant (HCT).^1,2^ However, in 2022, the Society for Healthcare Epidemiology of America (SHEA) published guidelines discouraging routine use of this strategy, given its limited utility in preventing nosocomial transmission, along with the logistical challenges and costs of testing.^3^ The utility of screening of asymptomatic patients to prevent transmission among those at highest risk for COVID-19-related morbidity and mortality, such as patients undergoing HCT or chimeric antigen receptor T-cell therapy (CAR T-cell), has not been well characterized.^1^ This study examines the utility of SARS-CoV-2 screening among asymptomatic HCT and CAR T-cell recipients at a large comprehensive cancer center by measuring positivity rates, false positives, treatment delays, and days in isolation during a time in which weekly screening tests were routinely employed.

## Methods

### Study design and data collection

This is a single-center retrospective cohort study of patients aged ≥ 18 years who presented for HCT or CAR T-cell therapy after April 30^th^, 2021, and underwent therapy before March 1^st^, 2023. The cohort was identified using a center-specific electronic database. Test indications and results were extracted for all SARS-CoV-2 nasopharyngeal and nasal Real-Time Polymerase Chain Reaction (RT-PCR) tests performed from patients’ initial visits with the HCT or CAR T-cell treatment services until either discharge from the service, day before second HCT or CAR T-cell infusion, death, date of last contact, or March 1, 2023, whichever came first.

During the study period, screening of asymptomatic patients was routinely conducted in the outpatient setting once patients established care with the HCT or CAR T-cell services and weekly thereafter. In the inpatient setting, screening occurred upon admission and weekly thereafter. Screening was also performed prior to hospital admissions or procedures. SARS-CoV-2 RT-PCR tests performed for either symptomatic or asymptomatic indications were eligible for analysis. Among positive tests, only the first positive test per patient was included.

Chart review of patients with a positive result and asymptomatic test indication was conducted by two independent reviewers to confirm or reclassify test indication, identify false-positive results, determine if and when symptoms were first noted, identify delays in procedures or treatment based on care team notations, and determine the length of isolation. Evaluation for source of acquisition was beyond the scope of this study. Discordant findings from the reviewers were adjudicated by a third independent reviewer. The study was approved by the center’s institutional review board

### Definitions and laboratory methods

False positives were identified through chart review and defined as patients who remained asymptomatic and had at least one negative repeat SARS-CoV-2 RT-PCR test in the day after the initial positive; Those who remained asymptomatic and did not have repeat testing were assumed to be true positives. Procedures were defined as medical interventions related to HCT or CAR T-cell therapy (eg, port placement, pulmonary function testing). Treatment was defined as therapies associated with cancer care, including chemotherapy, HCT, or CAR T-cell therapy. Samples were tested by RT-PCR as previously described.^4^ Isolation was defined as the use of transmission-based precautions (eg, personal protective equipment, special room requirements) to prevent the spread of SARS-CoV-2.

### Statistical methods

The primary outcome was the proportion of SARS-CoV-2 tests that were positive. Secondary outcomes included false positive results, delays in procedures and treatments, and days in isolation. Outcomes were described using frequency tabulations for categorical variables and medians and ranges for continuous variables. SAS version 9.4 (SAS Institute, Inc., Cary, NC) was used for all analyses.

## Results

**Figure 1.**
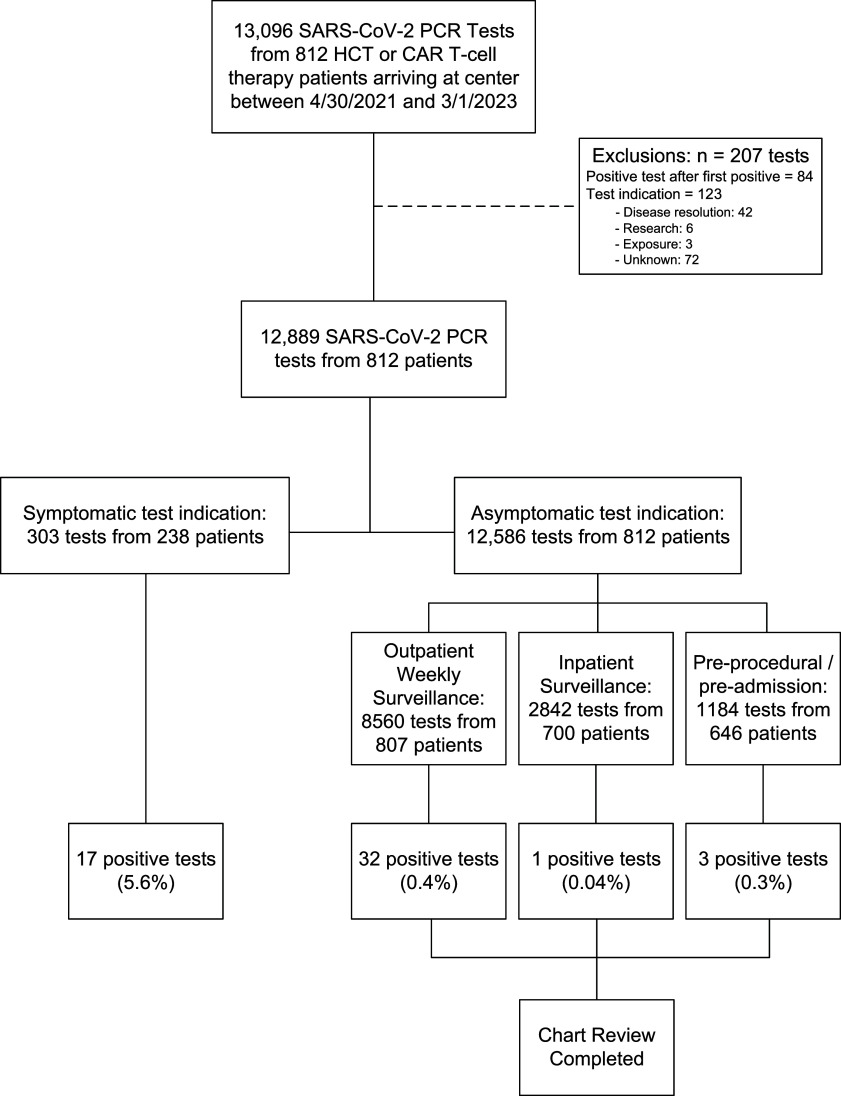
Study flowchart demonstrating a total of 12,889 SARS-CoV-2 RT-PCR tests from 812 patients were available to evaluate the primary outcome of percentage of tests that were positive. Thirty-six patients testing positive from screening testing were available to evaluate secondary outcomes of false positives, delays in cancer care, and length of isolation, determined from chart review.

Of 13,096 SARS-CoV-2 RT-PCR tests performed during the study period, 12,889 tests among 812 (650 HCT, 162 CAR T-cell) patients were eligible for analysis (Figure 1). The median number of tests per patient was 14 (range 1 – 51) for screening and 1 (range 1 – 10) for symptomatic testing. Positive tests were more frequent among those tested for symptoms (17/303, 5.6%) than those tested for screening (36/12,586, .3%). The percentage of positive tests was similar between HCT and CAR T-cell therapy recipients and between pre- and post-therapy time periods. (Supplemental Table 1).

**Figure 2.**
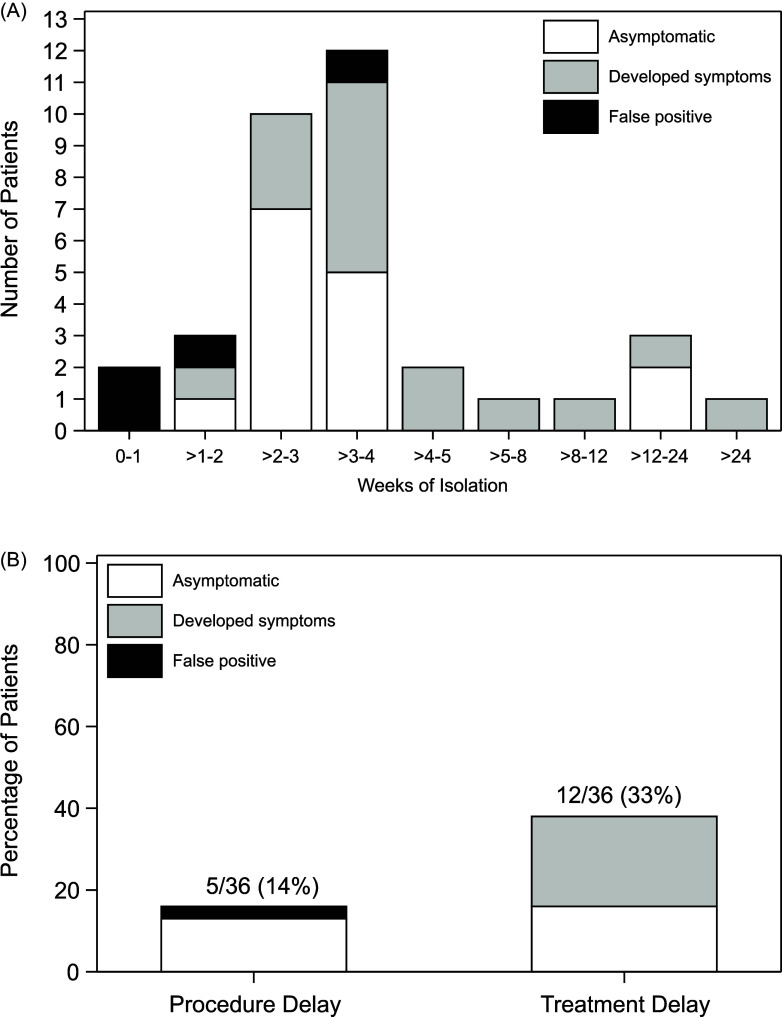
(A) Histogram showing the distribution of weeks of isolation among 35 patients testing positive for SARS-CoV-2 from screening testing of asymptomatic patients with at least one day of isolation. Empty bars represent patients with asymptomatic infection (*n* = 15), light gray bars represent those who developed symptoms (*n* = 16), and black bars represent false positives (*n* = 4). (B) Percentage of patients with delays in procedures or treatment among 36 patients testing positive for SARS-CoV-2 from screening testing. Empty bars represent patients with asymptomatic infection (4 with procedure delay, 5 with treatment delay), light gray bars represent those who developed symptoms (0 with procedure delay, 7 with treatment delay), and black bars represent false positives (1 with procedure delay, 0 with treatment delay). Two patients had delays in both procedures and treatments; 15/36 (42%) had delays in either procedures or treatments.

Among the 36 patients testing positive by screening, 16/36 (44%) went on to develop symptoms consistent with COVID-19, 15 (42%) remained asymptomatic, and 5 (14%) were false positives. Nearly all positive patients (35/36) were isolated. Among those who were isolation, the median duration of isolation was 22 days (range 2 – 266 days), with longest isolation periods among the symptomatic patients and shortest isolation periods among false positive patients (Supplemental Table 2, Figure 2a).

A total of 15/36 (42%) patients had delays in either procedures or treatments. Delays in procedures occurred among 5/36 (14%) patients, including one patient with a false positive result; delays in treatments, including HCT or CAR T-cell therapy, occurred in 12/36 (33%) patients (Supplemental Table 2, Figure 2b).

## Discussion

This study demonstrates the limited value of screening in high-risk patients undergoing HCT or CAR T-cell therapy with low yield of true positives (.2%) despite high rates of positivity in the community. Furthermore, among asymptomatic, true positive patients, half went on to develop symptoms consistent with COVID-19, suggesting identification would have occurred via symptom-based testing. While this study was not designed to measure the benefits of surveillance testing in terms of the impact on antiviral therapy or downstream transmission events, the rarity of true positive tests suggest a limited benefit. However, the prolonged isolation and delayed in care, both of which were noted in our study, suggest that overall, the risk likely outweighted the benefit.

While isolation may be perceived as a benign patient safety intervention, transmission-based precautions can significantly impact clinic workflow and patient care. Facility protocols may limit infectious persons in clinic, reschedule procedures to the end of the day, and convert in-person visits to telehealth, all of which may increase operational strain on clinics and contribute to patient dissatisfaction.^5,6^ Alternative interventions to limit transmission, such as universal masking, respiratory symptom screening, low provider threshold to test for symptoms, ready access to affordable testing, and vaccination likely provide greater benefit with less cost and fewer negative impacts to patients.

Study limitations include only SARS-CoV-2 RT-PCR results performed at our cancer center were included for analysis. While antigen tests were available during the study period, these were not routinely used by the center. Additionally, while two independent reviewers verified test indications for positive tests, the same did not occur for negative tests. Therefore, the electronic order indications among negative tests may not have represented the true asymptomatic or symptomatic clinical indication. Furthermore, as the population was limited to active HCT or CAR T-cell patients, a uniquely vulnerable population, results may not be generalizable. Moreover, it is possible that more false positives existed, as positive results with potential to impact cancer care were more likely to be followed up on with repeat testing to evaluate for false positivity than those without impact to the care plan. Treatment of COVID-19 infections was outside the scope of this study but has been described elsewhere.^7^ Finally, this study did not evaluate the value of screening in the presence of infection clusters or outbreaks.

This study of screening in asymptomatic HCT and CAR T-cell therapy patients demonstrates its limited value in mitigating SARS-CoV-2 transmission in non-outbreak settings, along with its potential to cause delays in cancer care. Rather than implementing high-cost, low-yield screening of asymptomatic individuals, future prevention efforts should focus on early symptomatic detection, readily available testing, and proper adherence to core infection prevention measures to protect high-risk populations.

## Supporting information

10.1017/ash.2025.10112.sm001 Wilson et al. supplementary material Wilson et al. supplementary material

## Supplementary material

The supplementary material for this article can be found at https://doi.org/10.1017/ash.2025.10112.
