# Supplementary material for: Limited yield of SARS-CoV-2 screening in asymptomatic hematopoietic cell transplant and chimeric antigen receptor T-cell therapy patients
Source: Antimicrob Steward Healthc Epidemiol. 2025 Aug 29;5(1):e195. doi: 10.1017/ash.2025.10112 (PMC12415789; doi:10.1017/ash.2025.10112)
Supplement: Wilson et al. supplementary material [file S2732494X25101125sup001.docx]

# **Supplemental Results**

| **Supplemental Table 1. Positive Test Percentages by Test Indication, Time Period, and Patient Group^1^** | | | | |
| --- | --- | --- | --- | --- |
|  |  | **Pre-HCT/CAR T-cell** | **Post-HCT/CAR T-cell** | **Pre- or Post** |
| **Patient Group** | **SARS-CoV-2 Test Indication** | **Number of positive tests / number of tests (%)** | **Number of positive tests / number of tests (%)** | **Number of positive tests / number of tests (%)** |
| **HCT Patients** |  |  |  |  |
|  | Asymptomatic outpatient weekly surveillance | 13 / 3654 (0.36%) | 12 / 3778 (0.32%) | 25 / 7432 (0.34%) |
|  | Asymptomatic inpatient surveillance | 0 / 562 (0.00%) | 1 / 1842 (0.05%) | 1 / 2404 (0.04%) |
|  | Asymptomatic pre-procedural/pre-admission | 1 / 580 (0.17%) | 2 / 408 (0.49%) | 3 / 988 (0.30%) |
|  | Symptomatic | 6 / 85 (7.06%) | 8 / 173 (4.62%) | 14 / 258 (5.43%) |
| **CAR T-cell Patients** |  |  |  |  |
|  | Asymptomatic outpatient weekly surveillance | 5 / 713 (0.70%) | 2 / 415 (0.48%) | 7 / 1128 (0.62%) |
|  | Asymptomatic inpatient surveillance | 0 / 84 (0.00%) | 0 / 354 (0.00%) | 0 / 438 (0.00%) |
|  | Asymptomatic pre-procedural/pre-admission | 0 / 81 (0.00%) | 0 / 115 (0.00%) | 0 / 196 (0.00%) |
|  | Symptomatic | 1 / 13 (7.69%) | 2 / 32 (6.25%) | 3 / 45 (6.67%) |
| **HCT and CAR T-cell Patients** |  |  |  |  |
|  | Asymptomatic outpatient weekly surveillance | 18 / 4367 (0.41%) | 14 / 4193 (0.33%) | 32 / 8560 (0.37%) |
|  | Asymptomatic inpatient surveillance | 0 / 646 (0.00%) | 1 / 2196 (0.05%) | 1 / 2842 (0.04%) |
|  | Asymptomatic pre-procedural/pre-admission | 1 / 661 (0.15%) | 2 / 523 (0.38%) | 3 / 1184 (0.25%) |
|  | Symptomatic | 7 / 98 (7.14%) | 10 / 205 (4.88%) | 17 / 303 (5.61%) |
| ^1^Among HCT and CAR T-cell patients establishing care with the HCT or CAR T-cell treatment service between 4/30/21 and 3/1/23 and SARS-CoV-2 test data. Excludes repeat positives after first positive for each patient. | | | | |

| **Table 2. Summaries Among Patients Testing Positive Under Asymptomatic Testing, by Time Period, and Patient Group^1^** | | | | |
| --- | --- | --- | --- | --- |
|  |  | **Pre-HCT/CAR T cell** | **Post-HCT/CAR T cell** | **Pre- or Post** |
| **Patient Group** | **Summary** |  |  |  |
| **HCT Patients** |  |  |  |  |
|  | Number of Patients | 14 | 15 | 29 |
|  | False positive result | 2 (14.3%) | 3 (20.0%) | 5 (17.2%) |
|  | Any delay in procedure | 4 (28.6%) | 1 (6.7%) | 5 (17.2%) |
|  | Any delay in treatment | 10 (71.4%) | 1 (6.7%) | 11 (37.9%) |
|  | Number of isolation days, median (range)^2^ | 21 (3, 28) | 22 (2, 266) | 22 (2, 266) |
|  | Any symptoms reported | 5 (35.7%) | 8 (53.3%) | 13 (44.8%) |
|  | Days from positive test to first symptom report, median (range) | 1 (0, 2) | 0 (-1, 11) | 0 (-1, 11) |
| **CAR T cell Patients** |  |  |  |  |
|  | Number of Patients | 5 | 2 | 7 |
|  | False positive result | 0 (0.0%) | 0 (0.0%) | 0 (0.0%) |
|  | Any delay in procedure | 0 (0.0%) | 0 (0.0%) | 0 (0.0%) |
|  | Any delay in treatment | 1 (20.0%) | 0 (0.0%) | 1 (14.3%) |
|  | Number of isolation days, median (range)^2^ | 21 (21, 52) | 103 (83, 123) | 31 (21, 123) |
|  | Any symptoms reported | 2 (40.0%) | 1 (50.0%) | 3 (42.9%) |
|  | Days from positive test to first symptom report, median (range) | 2 (1, 3) | 1 (1, 1) | 1 (1, 3) |
| **HCT and CAR T cell Patients** |  |  |  |  |
|  | Number of Patients | 19 | 17 | 36 |
|  | False positive result | 2 (10.5%) | 3 (17.6%) | 5 (13.9%) |
|  | Any delay in procedure | 4 (21.1%) | 1 (5.9%) | 5 (13.9%) |
|  | Any delay in treatment | 11 (57.9%) | 1 (5.9%) | 12 (33.3%) |
|  | Number of isolation days, median (range)^2^ | 21 (3, 52) | 24 (2, 266) | 22 (2, 266) |
|  | Any symptoms reported | 7 (36.8%) | 9 (52.9%) | 16 (44.4%) |
|  | Days from positive test to first symptom report, median (range) | 1 (0, 3) | 0 (-1, 11) | 1 (-1, 11) |
| ^1^Among HCT and CAR T cell patients with arrival date between 4/30/21 and 3/1/23 and SARS-CoV-2 test data. Includes 32 positive patients from asymptomatic weekly testing, 1 positive patient from inpatient asymptomatic testing, and 3 patients from asymptomatic pre-procedural/pre-admission testing. Numbers are n(%) unless otherwise specified. | | | | |
| ^2^Among 35 patients with any isolation days. | | | | |
